# Differential Microbial Signature Associated With Benign Prostatic Hyperplasia and Prostate Cancer

**DOI:** 10.3389/fcimb.2022.894777

**Published:** 2022-07-05

**Authors:** Purandar Sarkar, Samaresh Malik, Anwesha Banerjee, Chhanda Datta, Dilip Kumar Pal, Amlan Ghosh, Abhik Saha

**Affiliations:** ^1^ School of Biotechnology, Presidency University, New Town, Kolkata, India; ^2^ Department of Life Sciences, Presidency University, Kolkata, India; ^3^ Department of Pathology, Institute of Post Graduate Medical Education and Research, Kolkata, India; ^4^ Department of Urology, Institute of Post Graduate Medical Education and Research, Kolkata, India

**Keywords:** prostate cancer, benign prostate hyperplasia, microbiome, EBV, HPV

## Abstract

Apart from other risk factors, chronic inflammation is also associated with the onset of Prostate Cancer (PCa), wherein pathogen infection and tissue microbiome dysbiosis are known to play a major role in both inflammatory response and cancer development. However, except for a few studies, the link between microbes and PCa remained poorly understood. To explore the potential microbiome signature associated with PCa in Indian patients, we investigated differential compositions of commensal bacteria among patients with benign prostatic hyperplasia (BPH) and PCa using 16S rRNA amplicon sequencing followed by qPCR analyses using two distinct primer sets. Using two independent cohorts, we show that *Prevotella copri*, *Cupriavidus campinensis*, and *Propionibacterium acnes* represent the three most abundant bacteria in diseased prostate lesions. LEfSe analyses identified that while *Cupriavidus taiwanensis and Methylobacterium organophilum* are distinctly elevated in PCa samples, *Kocuria palustris* and *Cellvibrio mixtus* are significantly enriched in BPH samples. Furthermore, we identify that a number of human tumor viruses, including Epstein-Barr virus (EBV) and hepatitis B virus (HBV), along with two high-risk human papillomaviruses - HPV-16 and HPV-18, are significantly associated with the PCa development and strongly correlated with PCa bacterial signature. The study may thus offer to develop a framework for exploiting this microbial signature for early diagnosis and prognosis of PCa development.

## Introduction

Prostate cancer (PCa) is the second most common cancer and the sixth leading cause of cancer-associated deaths among men, leading to a great public health concern worldwide (Katongole et al., 2020). Due to increased life expectancy, adoption of newer lifestyles, and changes in food habits, PCa incidence is gradually increasing in India (Hariharan and Padmanabha, 2016). Increasing evidence suggests that chronic inflammation plays a vital role in PCa initiation and progression (Sfanos et al., 2017; Porter et al., 2018). The prostate gland harbors a relatively higher number of lymphocytes and other immune cells for protecting it from invading pathogens (Nickel and Xiang, 2008; Sfanos et al., 2017). While prostate-specific antigen (PSA) testing followed by trans-urethral resection of the prostate has long been recommended (Mottet et al., 2020), through this method, PCa detection at an early stage is not always accompanied by accurate determination of morbidity risk and therefore mortality might result due to over-treatment in some cases and under-treatment in others. Thus, a great attention has been made to explore better molecular approaches for the early diagnosis and prognosis of PCa.

Both oncogenic viruses and bacterial infections are thought to play critical roles in prostatic inflammation (Massari et al., 2019). A growing body of evidence suggests that several bacterial species including *E. coli* and other species of Enterobacteriaceae promote prostatic inflammation (Sfanos et al., 2017; Jain et al., 2020). The pro-inflammatory *Propionibacterium* species, particularly human skin associated *P. acnes*, has been well studied in connection to PCa, and has also been found to induce prostatic inflammation in animal models (Cohen et al., 2005; Olsson et al., 2012; Mak et al., 2013; Shinohara et al., 2013). Beside tissue microbiome dysbiosis, tumor virus infections are also shown to play a major role in cancer development, accounting for approximately 20% of all human cancers (Saha et al., 2010). Studies have shown that a number of viruses including multiple subtypes of human papillomaviruses (HPVs), polyomaviruses such as John Cunningham virus (JCV), BK virus (BKV), and simian virus 40 (SV40), as well as herpesviruses like human cytomegalovirus (HCMV) and Epstein-Barr virus (EBV), are associated with PCa specimens (Samanta et al., 2003; Das et al., 2008; Whitaker et al., 2013; Smelov et al., 2016; Banerjee et al., 2019).

Recently a number of next-generation sequencing as well as hybridization-based microarray studies identified potential microbiome signature associated with PCa development (Chen and Wei, 2015; Cavarretta et al., 2017; Yow et al., 2017; Banerjee et al., 2019). Despite these efforts, the pathogenic microbial composition of PCa may vary among the population of different ethnicities across the globe. Given the increasing incidence of PCa patients in India (Jain et al., 2014), a detailed and comprehensive analysis of the microbial ecosystem coupled with the PCa development is of utmost importance. Herein, we aim to identify specific microbial signature associated with the pathologic prostate tissue specimens collected from patients in the Eastern region of India.

## Materials and Methods

### Ethics Statement

The study was approved by the Institutional Review Board of Institute of Post Graduate Medical Education & Research (IPGME&R), Kolkata, India. Written informed consent was obtained from all participants and all methods in this study were performed in accordance with the ethical principles founded in the Declaration of Helsinki.

### Subject Recruitment and Specimen Collection

Affected individuals were recruited at Dept. of Urology, IPGME&R, Kolkata. A standard TRUS-guided 18-core prostate biopsy with subsequent histopathological analysis was carried out in those with abnormal serum PSA level (>4.0 ng/ml). All cases included were newly diagnosed patients without any prior treatment before surgery. Individuals with clinical evidence of prostatic lesions receiving radiotherapy and/or undergoing androgen deprivation therapy (ADT) were excluded. Digital images of biopsy slides were reviewed by genitourinary oncology pathologist. Samples were collected into two cohorts. Cohort-1 (Discovery Cohort) containing 13 BPH and 33 PCa tissue biopsy samples were subjected to 16S rRNA amplicon sequencing. Cohort-2 (Validation Cohort) containing 15 BPH and 16 PCa tissue biopsy samples together with Cohort-1 were used for validation using real-time qPCR analyses. Clinical characteristics and other information related to both sample cohorts are described in **Table S1**.

### Nucleic Acid Extraction

A total of 77 formalin-fixed paraffin-embedded (FFPE) tissue samples in both cohorts including 28 BPH and 49 PCa tissue biopsy specimens were received as 10 µm sections on non-charged glass slides. Genomic DNA was isolated according to the standard procedure as previously described (Mitra et al., 2010). Isolated genomic DNA was stored at -20°C for future use. The quality and quantity of extracted DNA was determined by agarose gel electrophoresis and the A260/280 ratio using Synergy H1 Multimode Microplate Reader (BioTek Instruments, Inc., Winooski, VT, USA). Approximately 50 ng and 1 ng of DNA from each sample were used for 16S rRNA amplicon sequencing and real-time qPCR analyses, respectively.

### 16S rRNA Amplicon Sequencing and OTU Assignments

For characterization of bacterial populations, taxonomical analysis, and species identification, different hypervariable regions of 16S rRNA genes were amplified and sequenced on an Ion GeneStudio S5 System (Thermo Fisher Scientific Inc., Waltham, MA, USA) *using* Ion 550 Chip. Ion AmpliSeq Libraries were prepared using an automated Ion Chef system (Thermo Fisher Scientific Inc., Waltham, MA, USA) and subsequently placed in the Ion Chef System for emulsion PCR. The concentration and quality of the amplicons were measured using Agilent 2100 Bioanalyzer (Agilent Technologies, Santa Clara, CA, USA).

To assign taxonomy, each unique sequence was mapped using two comprehensive 16S rRNA reference databases – Greengenes v13.5 and the Thermo Fisher Scientific in-house MicroSeq 500 v2013.1. Reads were aligned against the databases using MegaBLAST. The expectation value (E-value) for the searches was set to 0.01, and the max target hits value was set to 100. To assign taxonomy, the minimum alignment percentage read to a subject sequence was set to a threshold of 90. A read was assigned to a genus only when the identity score of the sequence alignment was at 97% or higher. For species assignment, the minimum percentage identity of the alignment was set to 99%. The taxonomy distribution counts, or abundance derived from the clustered reads, was subsequently transformed into the relative abundance of the individual species.

### Diversity and Bacterial Enrichment Analyses

For statistical analysis, MicrobiomeAnalyst, a web-based tool, was utilized. QIIME (v1.9.0) was used to evaluate alpha diversity including Observed Species, Chao1, and Shannon indexes. While Observed Species and Chao1 indexes are the indicators of species richness, Shannon index evaluates species diversity. The difference of alpha diversity between groups was evaluated by Wilcoxon Rank-Sum Test using SPSS (version 22).

To compare microbial compositions between different groups, beta diversity was evaluated by calculating weighted UniFrac distances using Bray-Curtis method from the OTU abundance and utilized in Principal Component Analysis (PCoA). PERMANOVA algorithm on weighted UniFrac distance matrices for statistical significance between groups using 999 permutations in QIIME was applied to generate PCoA plots. The Differential abundance analysis between groups was performed using Metastats and *P*-values were adjusted for multiple hypotheses testing using the False Discovery Rate (FDR) based on the Benjamini-Hochberg procedure. The differential abundances of OTUs and specific OTU enrichment between different groups were determined using LEfSe based on Kruskal–Wallis H test. P-value and FDR were adjusted to 0.05. The unique bacterial composition among sample groups was identified using Random Forest classification algorithm within MicrobiomeAnalyst. Galaxy, an online tool for metagenomics analysis, was used to build the Cladogram.

### Functional Pathways Prediction

Functional compositions of the bacterial communities among different groups were predicted using Phylogenetic Investigation of Communities by Reconstruction of Unobserved States (PICRUSt) according to the Kyoto Encyclopedia of Genes and Genomes (KEGG) database using STAMP v2.1.3. P-value and FDR cutoff were adjusted to 0.05 level of significance.

### Real-Time qPCR Analyses

Gene-specific primers were designed using Primer-BLAST tool in NCBI database for real-time qPCR analyses and are listed in **Table S3**. qPCR primers were obtained from IDT, Inc. (Coralville, IA, USA). The optimum primer melting temperature (Tm) was set at 60°C and the maximum GC content was kept at 55%. qPCR analysis was performed using iTaq Universal SYBR Green Supermix (BIO-RAD, Hercules, CA, USA) in CFX Connect Real-Time PCR detection System (BIO-RAD, CA, USA) with the following thermal profile – one cycle: 95°C for 10 min; 40 cycles: 95°C for 10 s followed by 60°C for 10 s; and finally the dissociation curve at –95°C for 1 min, 55°C 10 s, and 95°C for 10 s. Unless otherwise stated, each sample was performed in duplicate and calculation was made using a -ΔCT method to quantify relative abundance compared with human genomic GAPDH control. The -ΔCt values of each sample were plotted using GraphPad Prism 8.0.1 for data output.

### Correlation Analysis Between Microbes

Correlation among bacteria and oncogenic viruses were analyzed using R software. Spearman’s rank test was performed using the ΔCt value of both bacteria and viruses. P-value cutoff was adjusted to 0.05 significance level.

## Results

### Sample Characteristics and Sequencing Data Summary of Diseased Prostate Samples

To understand the changes of microbial compositions associated with prostate cancer (PCa) development, we prospectively collected a total of 46 tissue biopsy samples from 13 benign prostatic hyperplasia (BPH) and 33 PCa patients (‘Discovery Cohort,’ **Table S1**). The total DNA of each specimen from the ‘Discovery Cohort’ was extracted and subjected to 16S rRNA amplicon-based sequencing on an Ion GeneStudio S5 System targeting the hypervariable regions of 16S rRNA gene *using* two distinct primer sets. While set-I primer was used for amplification of hypervariable regions including V2, V4, and V8, set-II primer was used for hypervariable regions including V3, V6-7, and V9. Additionally, as described later, to validate the sequencing data, we also collected another 31 tissue biopsy samples from 16 BPH and 15 PCa patients (‘Validation Cohort,’ **Table S1**).

A total of 35,320,332 raw reads were generated after sequencing of the 46 samples from the ‘Discovery Cohort’. The number of raw sequence reads varied by approximately 10 fold across samples. After quality trimming and chimera checking, 30,641,026 high-quality reads were mapped, while 6,301,956 reads were excluded due to low copy number reads (<10 copy numbers) and 41,312 reads were found to be un-mapped (**Table S2**). Subsequently, 24,297,758 mapped reads identified Operational Taxonomic Units (OTUs) at the level of family (>95% similarity index), genus (>97% similarity index), and species (>99% similarity index). Overall, a total of six phylum, 127 genera, and 291 bacterial species were identified from all 46 samples in the Discovery Cohort.

### Overall Bacterial Abundance and Diversity Among BPH and PCa Samples

**Figure 1:**
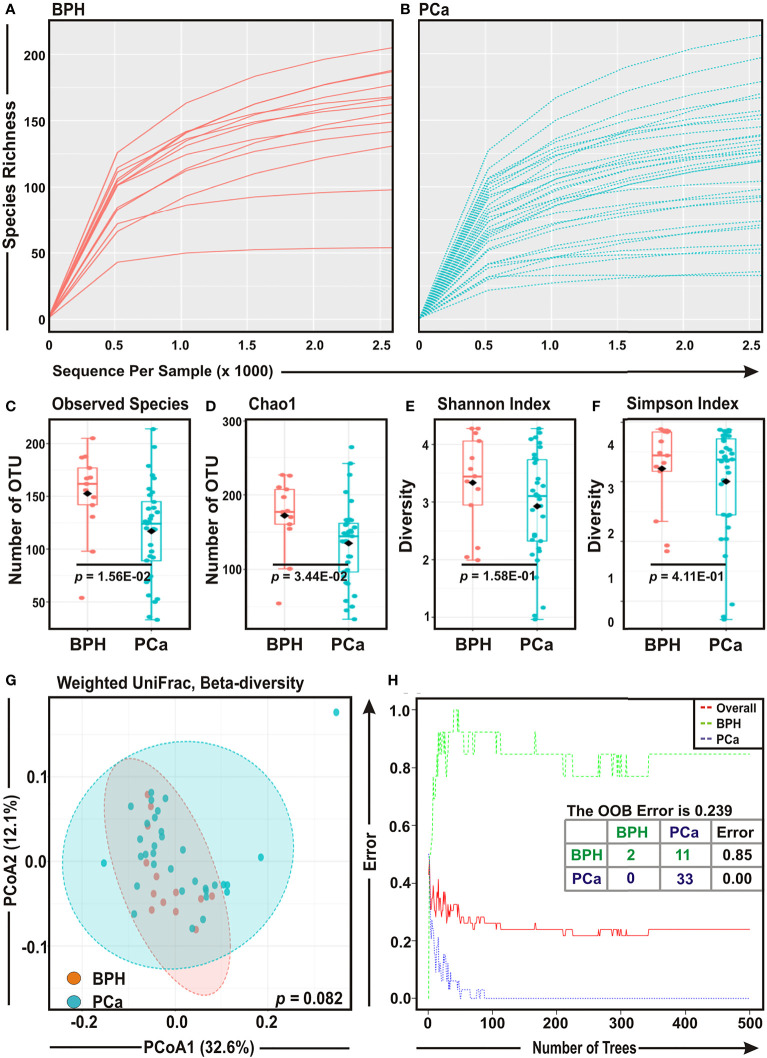
Comparison of prostate microbial ecology among benign prostatic hyperplasia (BPH) and prostate cancer (PCa) specimens. **(A, B)** Rarefaction analysis of bacterial 16S rRNA amplicon sequences of **(A)** 13 BPH (red) and **(B)** 33 PCa (blue) tissue biopsy samples. Each line represents one sample. **(C–F)** Box-Whisker plots of **(C)** Observed Species, **(D)** Chao 1, **(E)** Shannon Index, and **(F)** Simpson Index, respectively among BPH and PCa samples. **(G)** Principal Component Analysis (PCoA) plot based on weighted UniFrac distance matrices with respect to the bacterial abundance and composition among BPH and PCa tissue biopsy specimens. Axis 1 (PCoA1): 32.6% of variation explained. Axis 2 (PCoA2): 12.1% of variation explained. **(H)** The error plots identified from random forest classification analyses of BPH and PCa samples. Red-line indicates the overall species present in all samples including. While green-line indicates the distinct species present in BPH samples, purple-line indicates the specific species present in PCa lesions.

Rarefaction curves of species richness against sequences per sample were plotted for BPH and PCa samples to determine the efficiency of the sequencing process (**Figures 1A, B**). Most of the samples, though not completely, reached a saturated plateau phase, indicating that the depth of sequencing was sufficient for the diversity analysis (**Figures 1A, B**). Both Observed Species (*p* = 0.015) and Chao1 index (*p* = 0.034) showed that species richness was significantly decreased in PCa samples as compared to BPH samples (**Figures 1C, D**). In addition, the diversity estimators for both Shannon index (*p* = 0.158) and Simpson index (*p* = 0.411) indicated a trend of depletion in relative diversity of species composition in PCa samples as compared to BPH samples, although the data were not statistically significant (**Figures 1E, F**).

To assess the diversity among two groups, we evaluated weighted UniFrac distance matrix from the OTU abundance through utilizing PERMANOVA algorithm and subsequently applied in Principal Component Analysis (PCoA) (**Figure 1G**). Given that PCoA analyses revealed no significant difference (*p* = 0.082) in the bacterial compositions between BPH and PCa samples (**Figure 1G**), a ‘Random Forest’ algorithm was applied to further confirm the difference in bacterial community among the BPH and PCa biopsy samples (**Figure 1H**). The decision trees extracted from the random forest classification identified distinct bacterial composition in two BPH samples and 11 BPH samples exhibited overlapping species with PCa samples (class error: 0.850; **Figure 1H**). In contrast, all 33 PCa samples demonstrated unique bacterial compositions (**Figure 1H**).

### Taxonomiccharacterization of Bacterial Compositions Among BPH and PCa Samples

The bacterial communities associated with the diseased prostate lesions were further analyzed at different taxonomic levels. Six phyla including *Firmicutes*, *Proteobacteria*, *Bacteroidetes*, *Actinobacteria*, *Fusobacteria*, and *Deinococcus-Thermus* collectively comprised the entire sequences in both groups (**Figures S1A, B**). In general, *Proteobacteria* was the most abundant phylum in the prostate microbial ecology, contributing ~40.6% in diseased prostate lesions (**Figures S1A, B**). Overall, the results indicated that only *Actinobacteria* phylum was significantly depleted in PCa samples as compared to the BPH category (**Figures S1A, B**). At the genus level, *Prevotella*, *Cupriavidus*, *Propionibacterium*, *Acinetobacter*, and *Corynebacterium* represented the top five genera in both samples (**Figures S1C, D** and **S2A**). Of all genera detected, both groups shared approximately half of the total genera identified, i.e., 56/107 in BPH category and 56/118 in PCa lesions (**Figure S2B**).

To identify the differentially enriched genera within BPH and PCa samples, we employed Linear Discrimination Analysis (LDA) Effect Size (LEfSe) method (**Figure S1E**). LEfSe analysis of top 20 bacterial genera identified *Kocuria*, *Staphylococcus*, *Corynebacterium*, *Cellvibrio*, *Pseudomonas*, *Paracoccus*, *Brachybacterium*, *Pseudoxanthomonas*, *Anaerococcus*, *Stenotrophomonas*, *Microvirga*, *Empedobacter*, *Lysobacter*, *Brevibacterium*, *Comamonas*, *Serinicoccus*, *Rhodobacter*, *Chryseobacterium*, and *Aeromicrobium* as enriched genera in BPH samples, whereas only *Bradyrhizobium* genus was found to be significantly elevated in PCa samples (**Figure S1E**).

### Comparable Enrichment Analysis at Species Level Identified Significant Bacterial Compositions Between BPH and PCa Samples

**Figure 2:**
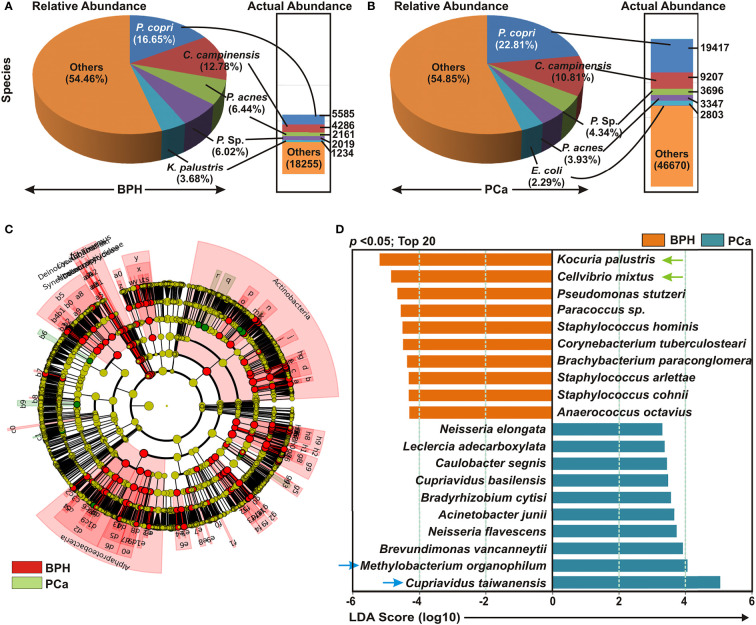
Cladogram and enrichment analysis of specific bacterial species among BPH and PCa lesions. **(A, B)** Relative and actual abundance of bacterial communities at the species levels in **(A)** BPH and **(B)** PCa tissue samples. **(C)** Cladogram derived from LEfSe analyses showing the taxonomic differences among BPH (red) and PCa (green) tissue biopsy specimens. **(D)** Association of specific microbiota taxa (top 20 bacterial species) with BPH and PCa samples by LEfSe analyses. Orange indicates taxa enriched in BPH and green indicates taxa enriched in PCa samples.

Detailed analyses of bacterial compositions at species level demonstrated *Prevotella copri*, *Cupriavidus campinensis*, *Propionibacterium acnes*, and Paracoccus sp. covered almost 50% of species variety in both BPH and PCa samples (**Figures 2A, B**). Next, cladogram and LEfSe analyses were conducted to further uncover the differential species compositions between BPH and PCa tissue biopsy samples (**Figures 2C, D**). The LDA scores demonstrated that among the top 20 most significantly enriched species *Kocuria palustris, Cellvibrio mixtus, Pseudomonas stutzeri, Paracoccus sp, Staphylococcus hominis*, *Corynebacterium tuberculostearicum*, *Brachybacterium paraconglomeratum*, *Staphylococcus arlettae*, *Staphylococcus cohnii*, and *Anaerococcus octavius* were notably elevated in the BPH samples, while *Cupriavidus taiwanensis*, *Methylobacterium organophilum*, *Brevundimonas vancanneytii*, *Neisseria flavescens*, *Acinetobacter junii*, *Bradyrhizobium cytisi*, *Cupriavidus basilensis*, *Caulobacter segnis*, *Leclercia adecarboxylata*, and *Neisseria elongata* were significantly increased in the PCa samples (**Figure 2D**).

### Validation of Species Identified in 16S rRNA Sequencing by Quantitative Real-Time PCR Analyses in BPH and PCa Samples

**Figure 3:**
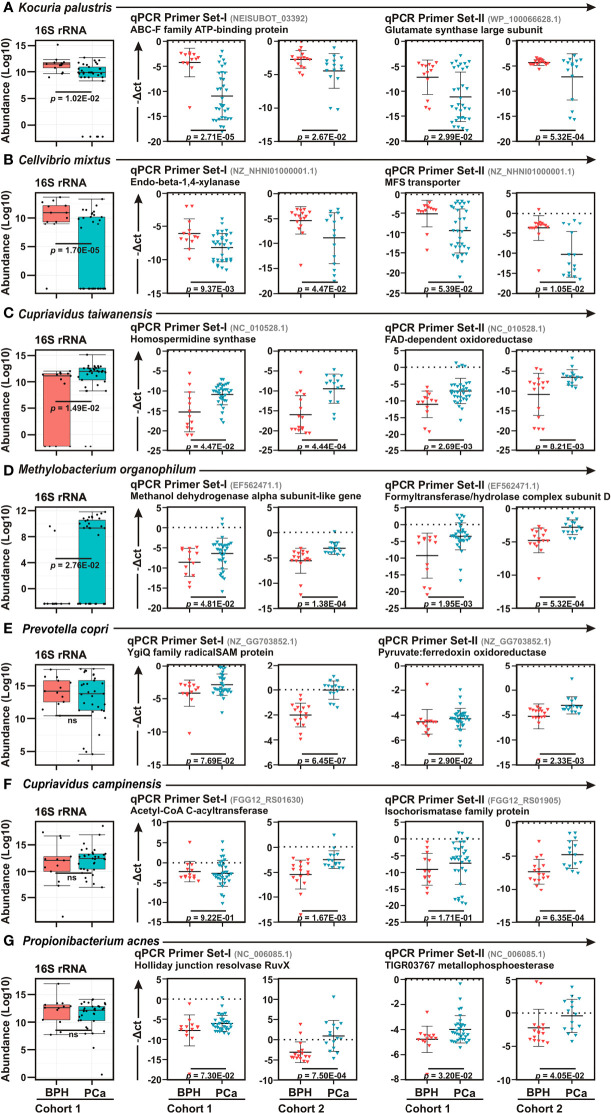
Real-time qPCR confirmation of bacterial species among BPH and PCa lesions. **(A–G)** Box Whisker plots (left panels) of species enrichment analysis of **(A–D)** top two bacterial species identified in LEfSe analysis as described in **Figure 3D** and **(E–G)** top three most abundant bacterial species as described in **Figures 3A, B** among BPH (orange) and PCa (green) tissue biopsy lesions of Cohort-1 16S rRNA sequencing results. **(A–G)** Relative qPCR analyses (right panels) of **(A)** *Kocuria palustris*, **(B)** *Cellvibrio mixtus*, **(C)** *Cupriavidus taiwanensis*, **(D)** *Methylobacterium organophilum* **(E)** *Prevotella copri*, **(F)** *Cupriavidus campinensis*, and **(G)** *Propionibacterium acnes* among BPH (orange) and PCa (green) samples of both Cohort-1 and Cohort-2 using two sets of primers. Specific gene primer is indicated on the top of each graph. Primers were designed using Primer-BLAST tool of NCBI web-portal. PCR calculation was performed by -ΔCT method to quantify relative abundance of each bacterium using human genomic GAPDH as control. The -ΔCt values of each sample were plotted using GraphPad Prism 8.0.1.

To confirm the 16S rRNA sequencing data, quantitative real-time PCR (qPCR) analyses were performed of top two bacteria in each category – BPH (*K. palustris* and *C. mixtus*) and PCa (*C. taiwanensis* and *Methylobacterium organophilum*), along with three most abundant bacteria (*P. copri*, *C. campinensis*, and *P. acnes*) identified in diseased prostate lesions, using two distinct species-specific primers (**Figure 3**). Moreover, in order to further corroborate the results, we used two sample cohorts – ‘Cohort-1’ (Discovery Cohort) and ‘Cohort-2’ (Validation Cohort) as described in **Table S1**. As similar to LEfSe analyses between BPH and PCa biopsy samples, qPCR data further confirmed *Kocuria palustris* and *Cellvibrio mixtus* as BPH specific and *Cupriavidus taiwanensis*, and *Methylobacterium organophilum* as PCa specific bacterial species in both sample cohorts (**Figures 3A, D**). In contrast to 16S rRNA sequencing results, qPCR analyses demonstrated *Prevotella copri*, *Cupriavidus campinensis*, and *Propionibacterium acnes* were significantly enriched in PCa samples as compared to the BPH group in both sample cohorts (**Figures 3E–G**).

### Functional Prediction of Altered Microbiome Associated With the PCa Development

In order to visualize the functional effects resulting from the altered microbial community associated with disease progression in prostate, we employed Phylogenetic Investigation of Communities by Reconstruction of Unobserved States (PICRUSt) software. PICRUSt analyses can predict the functional Kyoto Encyclopedia of Genes and Genomes (KEGG) pathways related to the composition of a metagenome and have been demonstrated to provide a good representation of metagenomic prediction. The LEfSe outputs of KEGG pathways among BPH and PCa tissue biopsy samples identified functions related to starch and sucrose metabolism, galactose metabolism, carbohydrate metabolism, primary immunodeficiency, ubiquitin system, Ion channels, proteasome, phenylpropanoid biosynthesis, electron transfer carriers, glycan degradation, and N-Glycan biosynthesis were significantly associated with BPH condition, while pathways such as nitrotoluene degradation, steroid hormone biosynthesis, non-homologous end-joining, and primary bile acid biosynthesis were directly linked to PCa development (**Figure S3A**). PCoA also demonstrated that the predicted functions of bacterial compositions among BPH and PCa were significantly clustered (p < 0.05) (**Figure S3B**).

### Microbial Dysbiosis Associated With Increasing Patient’s Age and PCa Grade

It is known that prostatic disease commonly affects middle-aged and elderly people (Perdana et al., 2016). In order to determine whether there is any link between age and microbial dysbiosis among BPH and PCa samples, correlation studies among the top 10 bacterial species identified in LEfSe analyses in each category with the patient’s age were further conducted (**Figures S4A, B**). While no correlation was established among BPH-specific bacteria with patient’s age, a number of bacterial species specific to PCa samples were moderately positively correlated with increasing patient’s age (**Figure S4B**). These include *Neisseria elongate* (r = 0.262), *Caulobacter segnis* (r = 0.277), and *Bradyrhizobium cytisi* (r = 0.346) (**Figure S4B**). Among the top two bacteria in LEfSe analyses along with the three most abundant bacteria in diseased prostatic lesion, *C. taiwanensis* (r = 0.176), *C. campinensis* (r = 0.162), and *P. acnes* (r = 0.191) were weakly positively correlated with PCa pateint’s age (**Figure S4C**). In contrast, while *C. taiwanensis* (r = -0.239) and *M. organophilum* (r = -0.131) were somewhat negatively correlated with PCa grade based on Gleason score, *P. copri* was found to positively correlated (r = 0.196) with PCa grade (**Figure S4D**).

### Quantitative Real-Time PCR Analyses Detected Strong Association of Multiple Human Tumor Viruses With PCa Progression

**Figure 4:**
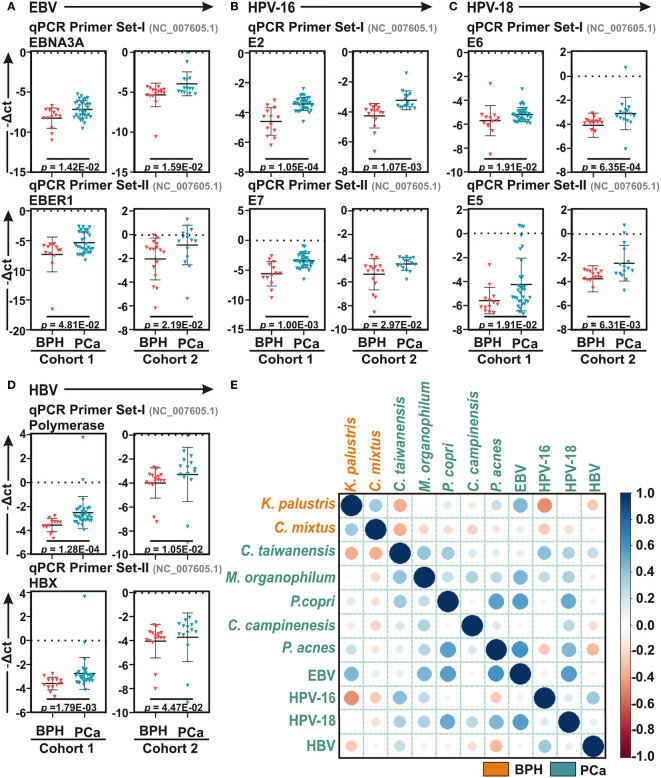
Association of human tumor viruses with BPH and PCa lesions. **(A–D)** Comparative qPCR data of **(A)** EBV, **(B)** HPV-16, **(C)** HPV-18 and **(D)** HBV among BPH and PCa samples of both Cohort-1 and Cohort-2 using two sets of primers. Specific gene primer is indicated on the top of each graph. Primers were designed using Primer-BLAST tool in NCBI database. PCR calculation was performed by -ΔCT method to quantify relative abundance of each tumor virus using human genomic GAPDH as control. The -ΔCt values of each sample were plotted using GraphPad Prism 8.0.1. **(E)** Co-occurrence and co-exclusion analyses between most abundant bacteria and human oncogenic viruses in BPH and PCa lesions. Pearson correlations among EBV, HPV-16, HPV-18, and HBV with the top two most abundant bacterial species identified in LEfSe and qPCR analyses in each group of BPH and PCa tissue biopsy samples were calculated and analyzed. Correlation values range from -1.0 (red) to +1.0 (blue). Orange: BPH specific; Green: PCa specific.

Studies suggest that a number of human oncogenic viruses including high-risk HPVs and EBV are associated with PCa development (Pascale et al., 2013; Whitaker et al., 2013; Smelov et al., 2016). To evaluate the potential involvement of viral etiology in our samples we designed qPCR primers for seven human tumor viruses including EBV, two high risk HPVs – HPV-16 and HPV-18 - HBV, HTLV-1, HCV, KSHV, and MCPyV along with two more human polyomaviruses – JCV and BKV (**Table S3**). qPCR analyses of Cohort-1 with primer set-I against all virus-specific antigens demonstrated that only EBV, HPV-16, HPV-18, and HBV are significantly associated with PCa samples as compared to BPH controls (**Figures 4A–D** and **S5A–F**). The housekeeping gene, human GAPDH gene, was utilized as control assuming the genomic segment bearing GAPDH gene remained unaffected in both BPH and PCa samples. A higher negative -ΔCt (average GAPDH Ct value – average target primer Ct value) indicated elevated presence of the virus in the sample as detected by specific primer set targeting specific viral gene. We further validated the association of these four selected tumor viruses in Cohort-2 using a second set of primers (**Figures 4A–D** and **Table S3**). The results clearly demonstrated that all four tumor viruses were significantly associated with the PCa lesions in comparison to BPH samples (**Figures 4A–D**).

### Co-Occurrence of Tumor Viruses With Microbiome Signature Linked to BPH and PCa Lesions

To further corroborate the connection of these tumor viruses with the identified microbial signature associated with BPH and PCa lesions, the co-occurrence and co-exclusion patterns of EBV, HPV-16, HPV-18, and HBV with the most abundant bacterial species identified in LEfSe and qPCR analyses in each category were further investigated (**Figure 4E**). As depicted in both LEfSe and qPCR analyses, BPH-specific bacteria *K. palustris* and *C. mixtus* and PCa-specific bacteria *C. taiwanensis* and *M. organophylum* were moderately correlated with each other (r = 0.362 and 0.394, respectively), indicating that they fell into two distinct groups (**Figure 4E**). Among BPH-specific bacteria, interestingly only *K. palustris* was positively correlated with EBV (r = 0.446) and as expected negatively correlated with HPV-16 (r = -0.461) and to a lesser extent with HBV (r = -0.263) (**Figure 4E**). Among PCa-specific bacteria, while *C. taiwanensis* was positively correlated with HPV-16 (r = 0.411) and to lesser extents with HPV-18 (r = 0.328) and HBV (r = 0.246), *M. organophylum* were positively correlated with EBV (r = 0.456) and to lesser extents with HPV-18 (r = 0.325) and HPV-16 (r = 0.261) (**Figure 4E**). Among the most abundant bacteria in both BPH and PCa tissue samples, all three species *P. copri*, *C. campinensis*, and *P. acnes* were in general found to be somewhat positively correlated with the PCa-specific bacteria but not with BPH-specific bacteria (**Figure 4E**). Among these three species *P. copri* and *P. acnes* were particularly strongly correlated (r = 0.543) (**Figure 4E**). In addition, while *P. copri* was positively correlated with both *C. taiwanensis* (r = 0.369) and to a lesser extent *M. organophylum* (r = 0.246), *C. campinensis* and *P. acnes* were correlated with only *M. organophylum* (r = 0.318, and 0.282, respectively) (**Figure 4E**). While *P. copri* and *P. acnes* were robustly correlated with two tumor viruses – EBV (r = 0.551 and 0.599, respectively) and HPV-18 (r = 0.509 and 0.444, respectively) - *C. campinensis* was weakly correlated with only HPV-18 (r = 0.279) (**Figure 4E**). Among the four tumor viruses, EBV and HPV-18 were positively correlated with each other (r = 0.551), whereas HPV-16 and HBV were grouped together (r = 0.370) (**Figure 4E**).

## Discussion

A growing body of evidence indicated microbial infection as one of the predominant risk factors for PCa development (Sfanos et al., 2008; Sutcliffe et al., 2011; Whitaker et al., 2013). In addition, several studies derived from 16S rRNA amplicon based sequencing, whole genome shotgun sequencing, as well as hybridization based microarray techniques have evidently documented microbiome dysbiosis associated with disease progression (Cavarretta et al., 2017; Yow et al., 2017; Banerjee et al., 2019; Massari et al., 2019). Despite the increasing incidence of PCa (Jain et al., 2014), to date, there are no reports describing the microbial dysbiosis associated with PCa development among Indian patients. Herein, we identified specific microbial signature including both alteration of tissue specific commensal bacteria along with infection status of several tumor viruses linked with the pathologic prostate tissue specimens collected from patients in the Eastern region of India.

Multiple studies suggested that an inflammatory microenvironment is involved in the development of PCa precursor lesions that promote tumor initiation (Kwon et al., 2014; Sfanos et al., 2017). Although microbial infections are considered as a mainstay of chronic inflammation, to date, no single microbe was identified as a direct contributor to PCa development. However, several bacterial species are known to induce prostatic inflammation (Sutcliffe et al., 2006; Nickel and Xiang, 2008; Sutcliffe et al., 2011; Sfanos et al., 2017). In addition, *Propionibacterium acnes*, a Gram-positive pro-inflammatory bacterium ubiquitously found in the human skin, has been detected with elevated levels in prostate tissue specimens from patients with prostatitis and PCa and can promote prostatic inflammation in both cell line and animal models (Sfanos and Isaacs, 2008; Sfanos et al., 2008; Fassi Fehri et al., 2011; Olsson et al., 2012; Shinohara et al., 2013). Moreover, *P. acnes* infection in prostate epithelial cells resulted in increased cell proliferation and anchorage-independent cell growth (Fassi Fehri et al., 2011), indicating the possibility of *P. acnes* as one of the major contributing inflammatory factors for PCa development. In addition, transcriptomic analyses using RNA-Seq data of human prostate samples collected from both Caucasian and Chinese patients demonstrated significant expression of *P. acnes* genes in PCa samples as compared to the matched controls (Chen and Wei, 2015). In agreement to this, our 16S rRNA amplicon sequencing results also demonstrated that *P. acnes* represented one of the most abundant bacterial species in diseased prostate specimens including both BPH and PCa. Moreover, our qPCR analyses further demonstrated that *P. acnes* was likely to be more associated with PCa lesions in comparison to BPH samples. In addition, due to anatomical proximity urinary microbiota has also been shown to influence prostate pathophysiology (Yu et al., 2015; Shrestha et al., 2018). Various studies profiled urinary microbiota of adult men, which include genera *Corynebacterium, Streptococcus, Veillonella, Prevotella, Anaerococcus, Propionibacterium, Finegoldia, Staphylococcus*, and *Lactobacillus* (Nelson et al., 2010; Dong et al., 2011). Another study suggested *the* prevalence of pro-inflammatory bacteria and uropathogens in the urinary tract of PCa patients (Shrestha et al., 2018). In agreement to these previous reports, our results also demonstrated *Prevotella*, *Propionibacterium*, and *Corynebacterium* among the top five genera in both BPH and PCa lesions.

Increasing evidence suggested that the gastrointestinal (GI) microbiome controls the efficacy of various cancer treatments including both chemotherapy and immunotherapy (Iida et al., 2013; Vetizou et al., 2015). Although, there are no published data, GI microbiome could influence immunotherapy response in case of PCa patients. Of note, recently, Sfanos et al. demonstrated a difference in GI microbiome compositions in men undergoing treatment with androgen deprivation therapies (ADT), which may influence treatment response to ADT or to subsequent treatments including immunotherapy (Sfanos et al., 2018). In agreement to this, our study also demonstrated significant enrichment of steroid hormone biosynthesis pathway in PCa as compared to BPH samples. *Prevotella copri* is a frequent resident of the GI microbiome, and its higher prevalence has been consistently reported in non-Westernized populations as well as patients with rheumatoid arthritis, a systemic autoimmune disease (Scher et al., 2013; Tett et al., 2019). Interestingly, our 16S rRNA sequencing results also demonstrated that *P. copri* was the most abundant species in both BPH and PCa lesions. In addition, qPCR analyses showed *P. copri* was significantly associated with PCa samples, indicating potential involvement of GI microbiota with PCa development possibly through modulating inflammatory response.

Intriguing evidence is emerging that indicates a potential association of several human oncogenic viruses with PCa (Samanta et al., 2003; Das et al., 2008; Lin et al., 2011; Chen and Wei, 2015; Smelov et al., 2016; Banerjee et al., 2019). For example, several HPV subtypes, polyomaviruses - JCV and BKV and herpesviruses including HCMV and EBV are associated with PCa across the globe (Samanta et al., 2003; Das et al., 2008; Lin et al., 2011; Whitaker et al., 2013; Anzivino et al., 2015; Smelov et al., 2016). Moreover, the frequency of co-infection of EBV and high-risk HPV-18 has been shown to be significantly higher in PCa as compared to benign and normal prostate samples (Whitaker et al., 2013). In agreement to this, our results also demonstrated that EBV was strongly correlated with HPV-18 but not with HPV-16 in PCa samples. A recent report by Ishiguro et al. using 30 non-cancer and 182 PCa tissue specimens suggested that HBV and HCV infection are not linked with PCa development in Japanese patients (Ishiguro et al., 2017). In contrast, using two different qPCR primer sets, our study for the first time demonstrated that HBV infection was significantly associated with PCa development. Importantly, a number of studies suggested that both HBV and HCV infections can promote androgen receptor (AR) signaling in hepatocellular carcinoma (HCC). Intriguingly, the gender preference of HCC differs between HBV and HCV related cases. The male predominance in HBV-linked HCC is significantly higher than that of HCV-linked HCC (El-Serag and Rudolph, 2007), indicating a potential link of AR-signaling and HBV infection. It has been demonstrated that HBV oncoprotein HBx induces AR-responsive gene expression in an androgen concentration dependent manner (Chiu et al., 2007; Yang et al., 2009). Targeting the AR signaling axis has been, over decades, the mainstay of PCa therapy (Karantanos et al., 2013; Tiwari et al., 2020). Although ADT using several specific AR inhibitors blocks further tumor growth for some patients, most patients develop resistance to the treatment and subsequently develop to castration-resistant PCa (CRPC) associated with poor prognosis (Cai et al., 2018). It would be fascinating to investigate the role of HBV infection in PCa progression. Altogether, it is still unclear whether the co-existence of these tumor viruses along with microbial dysbiosis acts as a promoter or bystander in PCa development. Nevertheless, in order to improve the treatment strategy as well as the development of diagnosis and prognosis markers, it is important, from the perspective of genetic etiology, to clarify the connection. Our study clearly revealed that *C. taiwanensis*, *M. organophylum* followed by *P. acnes* and *P. copri* were the most correlated bacterial species with the infection status of EBV and HPV-18 and, to lesser extents, HPV-16 and HBV in PCa tissue specimens.

In sum, our study demonstrated that dysbiosis of tissue specific microbiome is directly linked to prostate health and disease. Future in-depth investigations with larger sample cohorts of different socioeconomic and ethnic backgrounds are required to discern

Whether the microbiota and/or their metabolites can be considered as novel biomarkers and therapeutic targets for PCa in Indian scenario. The mechanisms by which the microbial dysbiosis along with infection of oncogenic viruses facilitate tumorigenesis can provide unique opportunities to explore the microbiome signature for diagnostic, preventive, as well as expansion of current therapeutic strategies against PCa onset and progression.

## Supplementary Information

Additional supporting information may be found online in the **Supporting Information** section.

## Data Availability Statement

The datasets presented in this study can be found in online repositories. The names of the repository/repositories and accession number(s) can be found in the article/**Supplementary Material**.

## Ethics Statement

The studies involving human participants were reviewed and approved by Post Graduate Medical Education & Research (IPGME&R), Kolkata, India. The patients/participants provided their written informed consent to participate in this study.

## Author Contributions

PS and AS wrote the main manuscript text. PS and SM performed bioinformatic analysis. PS, AB, and SM performed the experiments. DP collected the samples. CD performed histopathological analysis. AB and AG performed sampling. AS conceived, designed, and successfully sought funding for the study. All authors have read and approved the content of this manuscript.

## Funding

This study was supported by grants from Wellcome Trust/DBT India Alliance Intermediate Fellowship research grant [IA/I/14/2/501537], Department of Science and Technology (DST), Govt. of India [CRG/2018/001044] and Science & Technology and Biotechnology, Govt. of West Bengal [1798 (Sanc.)/ST/P/S&T/9G-5/2019] to AS. The funders had no role in study design, data collection and analysis, decision to publish, or preparation of the manuscript.

## Conflict of Interest

The authors declare that the research was conducted in the absence of any commercial or financial relationships that could be construed as a potential conflict of interest.

## Publisher’s Note

All claims expressed in this article are solely those of the authors and do not necessarily represent those of their affiliated organizations, or those of the publisher, the editors and the reviewers. Any product that may be evaluated in this article, or claim that may be made by its manufacturer, is not guaranteed or endorsed by the publisher.

## Supplementary Material

Supplementary Figure 1 Composition of bacterial communities across samples at the phylum and genus levels. **(A–D)** Relative abundance of bacterial communities at the **(A, B)** phylum and **(C, D)** genus levels in **(A–C)** BPH and **(B, D)** PCa tissue samples. **(E)** Association of specific microbiota taxa (top 20 bacterial genera) by LEfSe analyses in BPH and PCa samples. Orange indicates taxa enriched in BPH and green indicates taxa enriched in PCa.

Supplementary Figure 2 Differentially abundant bacterial genera among BPH and PCa tissue biopsy samples. **(A)** Relative abundance (%) of the taxa at the genus level in BPH and PCa tissue biopsy lesions. **(B)** Venn diagram depicts distinct and overlapped genera among BPH and PCa sample groups.

Supplementary Figure 3 Predicted functional pathways associated with BPH and PCa lesions. **(A)** LEfSe analyses of potential functional pathways associated with BPH (orange) and PCa (green) tissue biopsy samples. Functional compositions of the bacterial species among different samples groups were predicted using PICRUSt according to the KEGG database. P-value and FDR cutoff was adjusted to 0.05 level of significance. **(B)** Principal Component Analysis (PCoA) plot comparing the bacterial functions associated with BPH and PCa tissue biopsy samples. Axis 1 (PCoA1): 80.1% of variation explained. Axis 2 (PCoA2): 17.1% of variation explained.

Supplementary Figure 4 Correlations among BPH and PCa specific bacteria with patient’s age and cancer grade. **(A, B)** Pearson correlations among the top 10 bacterial species identified in LEfSe analyses in each group of **(A)** BPH and **(B)** PCa tissue biopsy samples with patient’s age were calculated and analyzed. **(C, D)** Pearson correlations among the top two bacterial species identified in LEfSe analyses in PCa samples along with three most abundant bacteria in both BPH and PCa samples with patient’s **(C)** age and **(D)** PCa grade were calculated and analyzed. Correlation values range from -1.0 (red) to +1.0 (blue). Orange: BPH specific; Green: PCa specific; Purple: most abundant bacteria in diseased prostate sample.

Supplementary Figure 5 qPCR analyses of tumor virus association with BPH and PCa samples. **(A–J)** Comparative qPCR data of **(A)** EBV, **(B)** HPV-16, **(C)** HPV-18, **(D)** HBV, **(E)** HCV, **(F)** KSHV, **(G)** HTLV-1, **(H)** MCPyV, **(I)** BKV, and **(J)** JCV among BPH and PCa samples of Cohort-1. Specific gene primer is indicated on the top of each graph. PCR calculation was performed by -ΔCT method to quantify relative abundance of each tumor virus using human genomic GAPDH as control. The -ΔCt values of each sample were plotted using GraphPad Prism 8.0.1.
