# Supplementary figures and images for: Differential Microbial Signature Associated With Benign Prostatic Hyperplasia and Prostate Cancer
Source: Front Cell Infect Microbiol. 2022 Jul 5;12:894777. doi: 10.3389/fcimb.2022.894777 (PMC9294280; doi:10.3389/fcimb.2022.894777)

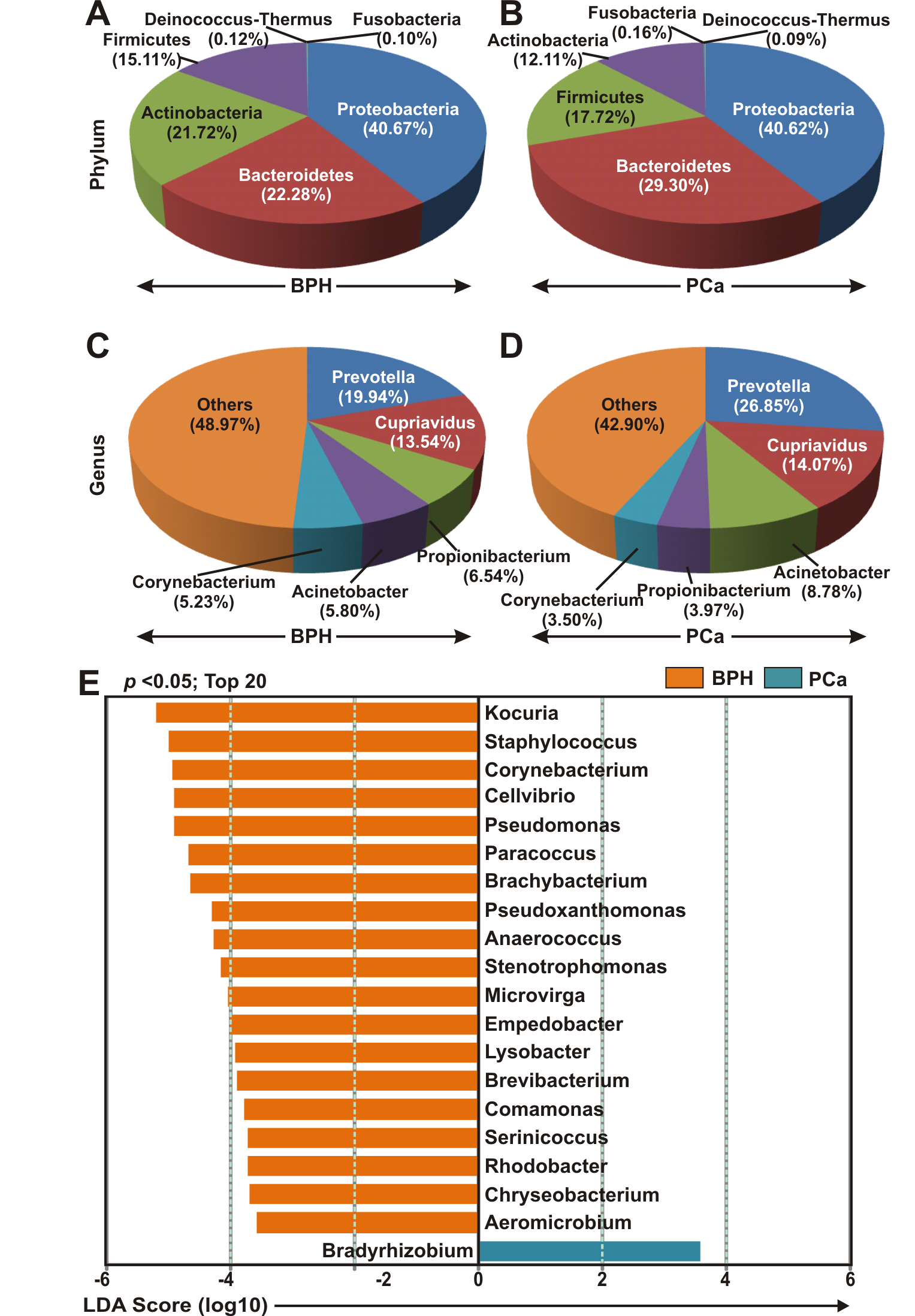

Supplement: Supplementary Figure 1 — Composition of bacterial communities across samples at the phylum and genus levels. (A–D) Relative abundance of bacterial communities at the (A, B) phylum and (C, D) genus levels in (A–C) BPH and (B, D) PCa tissue samples. (E) Association of specific microbiota taxa (top 20 bacterial genera) by LEfSe analyses in BPH and PCa samples. Orange indicates taxa enriched in BPH and green indicates taxa enriched in PCa. [file Image_1.tif]

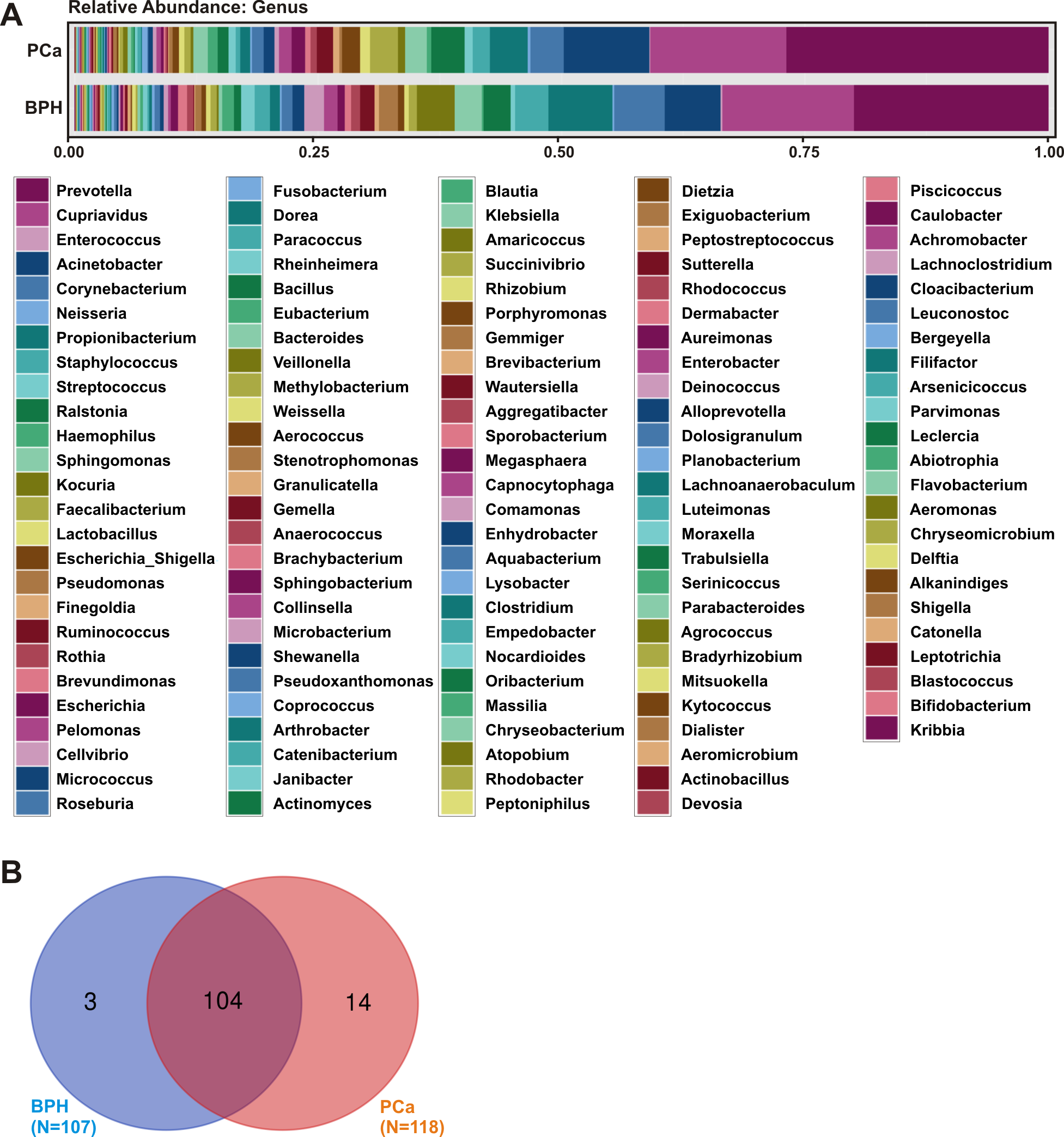

Supplement: Supplementary Figure 2 — Differentially abundant bacterial genera among BPH and PCa tissue biopsy samples. (A) Relative abundance (%) of the taxa at the genus level in BPH and PCa tissue biopsy lesions. (B) Venn diagram depicts distinct and overlapped genera among BPH and PCa sample groups. [file Image_2.tif]

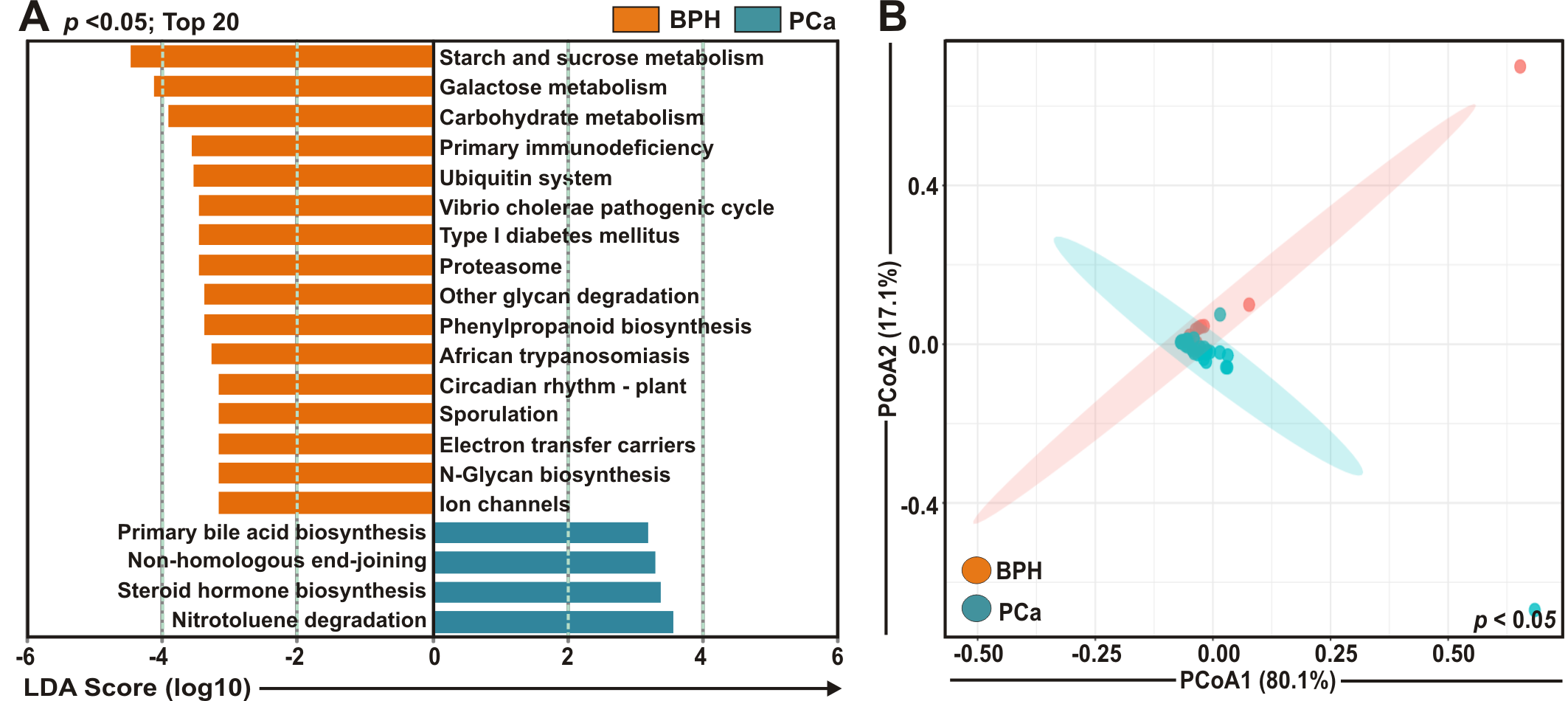

Supplement: Supplementary Figure 3 — Predicted functional pathways associated with BPH and PCa lesions. (A) LEfSe analyses of potential functional pathways associated with BPH (orange) and PCa (green) tissue biopsy samples. Functional compositions of the bacterial species among different samples groups were predicted using PICRUSt according to the KEGG database. P-value and FDR cutoff was adjusted to 0.05 level of significance. (B) Principal Component Analysis (PCoA) plot comparing the bacterial functions associated with BPH and PCa tissue biopsy samples. Axis 1 (PCoA1): 80.1% of variation explained. Axis 2 (PCoA2): 17.1% of variation explained. [file Image_3.tif]

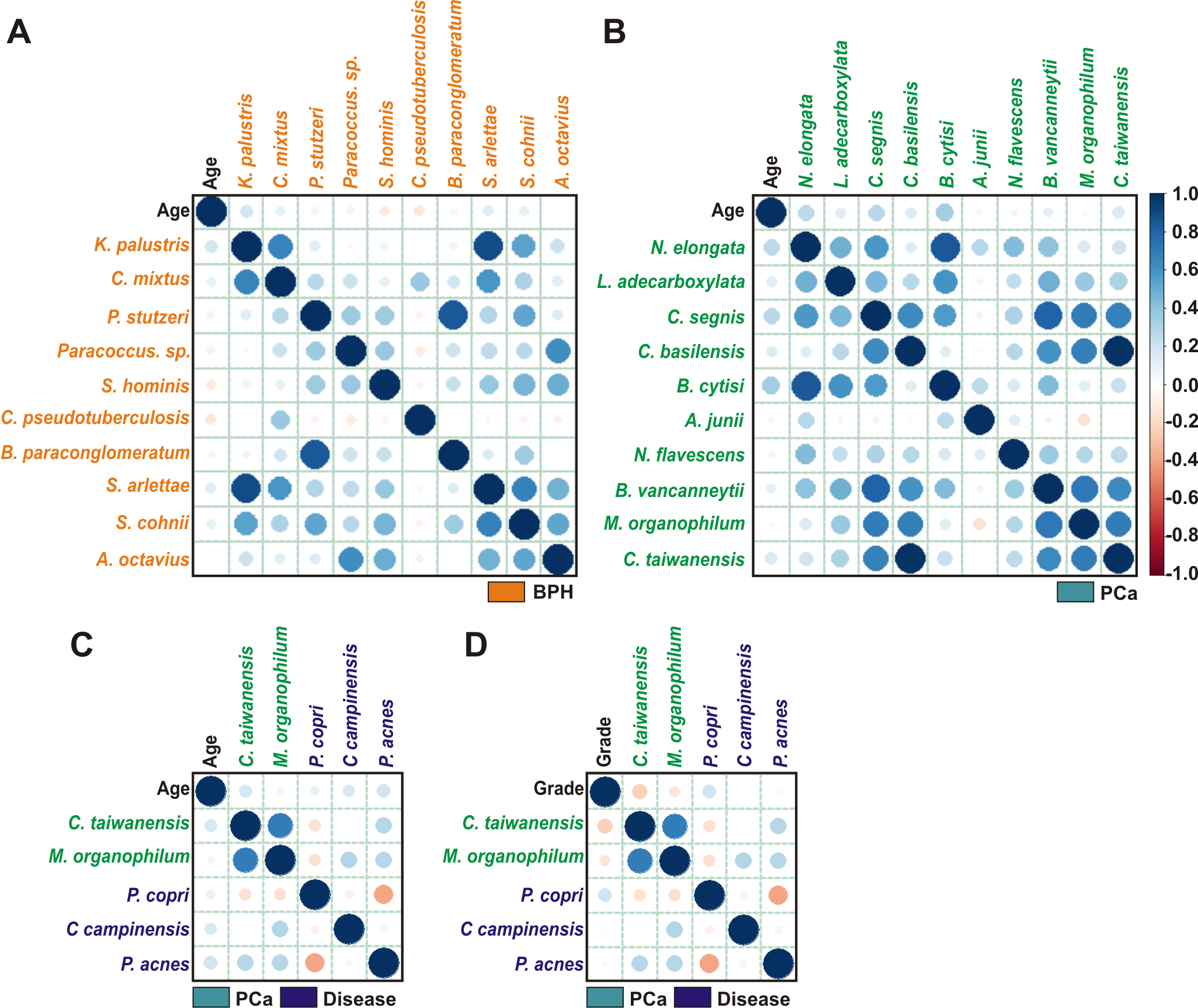

Supplement: Supplementary Figure 4 — Correlations among BPH and PCa specific bacteria with patient’s age and cancer grade. (A, B) Pearson correlations among the top 10 bacterial species identified in LEfSe analyses in each group of (A) BPH and (B) PCa tissue biopsy samples with patient’s age were calculated and analyzed. (C, D) Pearson correlations among the top two bacterial species identified in LEfSe analyses in PCa samples along with three most abundant bacteria in both BPH and PCa samples with patient’s (C) age and (D) PCa grade were calculated and analyzed. Correlation values range from -1.0 (red) to +1.0 (blue). Orange: BPH specific; Green: PCa specific; Purple: most abundant bacteria in diseased prostate sample. [file Image_4.tif]

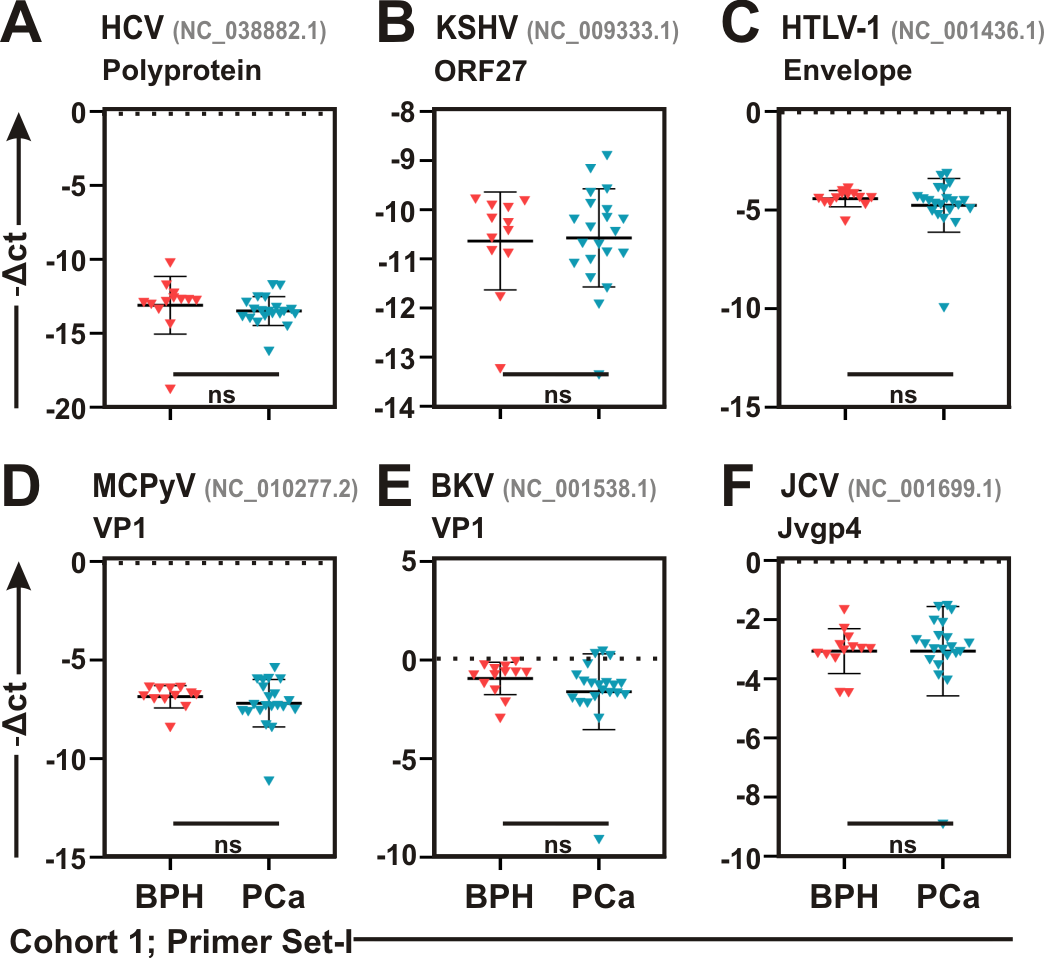

Supplement: Supplementary Figure 5 — qPCR analyses of tumor virus association with BPH and PCa samples. (A–J) Comparative qPCR data of (A) EBV, (B) HPV-16, (C) HPV-18, (D) HBV, (E) HCV, (F) KSHV, (G) HTLV-1, (H) MCPyV, (I) BKV, and (J) JCV among BPH and PCa samples of Cohort-1. Specific gene primer is indicated on the top of each graph. PCR calculation was performed by -ΔCT method to quantify relative abundance of each tumor virus using human genomic GAPDH as control. The -ΔCt values of each sample were plotted using GraphPad Prism 8.0.1. [file Image_5.tif]
