# Supplementary material for: Differential Microbial Signature Associated With Benign Prostatic Hyperplasia and Prostate Cancer
Source: Front Cell Infect Microbiol. 2022 Jul 5;12:894777. doi: 10.3389/fcimb.2022.894777 (PMC9294280; doi:10.3389/fcimb.2022.894777)
Supplement: Supplementary file 6 [file Table_1.docx]

**Table S1: Clinical characteristics of discovery and validation cohorts.**

|  | **Sample**  **ID** | | **Age**  **(yrs.)** | | **Sample types** | **PSA** | **Gleason score** | **Family**  **history of Cancer** | | **Smoking Habit** |
| --- | --- | --- | --- | --- | --- | --- | --- | --- | --- | --- |
|  | **Discovery Cohort (Cohort - 1)** | | | | | | | | | |
| **BPH***  (N = 13) | 75 | | 61 | | Biopsy | 2.92 | NA | No | | Yes |
|  | 82 | | 68 | | Biopsy | 6 | NA | No | | Yes |
|  | 87 | | 70 | | Biopsy | 8 | NA | No | | Yes |
|  | 89 | | 71 | | Biopsy | ND | NA | No | | Yes |
|  | 96 | | 56 | | Biopsy | 4.27 | NA | No | | Yes |
|  | 108 | | 60 | | Biopsy | 12.9 | NA | No | | Yes |
|  | 122 | | 60 | | Biopsy | 0.19 | NA | No | | No |
|  | 128 | | 46 | | Biopsy | 26.69 | NA | No | | No |
|  | 117 | | 60 | | Biopsy | 0.553 | NA | No | | No |
|  | 119 | | 60 | | Biopsy | 16.97 | NA | No | | No |
|  | 102 | | 77 | | Biopsy | 8 | NA | No | | No |
|  | 103 | | 80 | | Biopsy | 51.81 | NA | No | | No |
|  | 113 | | 78 | | Biopsy | 25.34 | NA | No | | No |
| **PCa#**  (N = 33) | 38 | | 63 | | Biopsy | 19.2 | 4+3=7/10 | No | | Yes |
|  | 183 | | 62 | | Biopsy | >1000 | 4+3=7/10 | No | | No |
|  | 143 | | 50 | | Biopsy | 162 | 3+4=7/10 | No | | Yes |
|  | 139 | | 78 | | Biopsy | 982 | 4+4=8/10 | No | | Yes |
|  | 58 | | 58 | | Biopsy | 145 | 3+4=7/10 | No | | Yes |
|  | 154 | | 72 | | Biopsy | 12.5 | 4+5=9/10 | No | | No |
|  | 92 | | 52 | | Biopsy | 157.4 | 3+4=7/10 | No | | Yes |
|  | 16 | | 65 | | Biopsy | 498 | 4+5=9/10 | No | | Yes |
|  | 135 | | 63 | | Biopsy | 3.9 | 5+4=9/10 | No | | No |
|  | 198 | | 62 | | Biopsy | NA | 3+4=7/10 | No | | No |
|  | 150 | | 56 | | Biopsy | 90 | 3+4=7/10 | No | | No |
|  | 100 | | 40 | | Biopsy | 980 | 5+5=10/10 | No | | No |
|  | 180 | | 78 | | Biopsy | 1777 | 3+4=7/10 | No | | No |
|  | 224 | | 64 | | Biopsy | 19.8 | 3+3=6/10 | No | | No |
|  | 93 | | 62 | | Biopsy | 57.08 | 3+3=6/10 | No | | Yes |
|  | 333 | | 80 | | Biopsy | 18.6 | 3+3 =6/10 | No | | No |
|  | 206 | | 60 | | Biopsy | 47.5 | 5+3=8/10 | No | | No |
|  | 131 | | 71 | | Biopsy | 68.4 | 4+3=7/10 | Father | | Yes |
|  | 202 | | 70 | | Biopsy | 0.8 | 4+4=8/10 | No | | No |
|  | 70 | | 73 | | Biopsy | 78.5 | 3+3=6/10 | No | | Yes |
|  | 134 | | 74 | | Biopsy | 85.75 | 3+3=6/10 | No | | Yes |
|  | 148 | | 55 | | Biopsy | 30.4 | 4+5=9/10 | No | | No |
|  | 327 | | 67 | | Biopsy | >100 | 4+3 =7/10 | No | | No |
|  | 141 | | 71 | | Biopsy | 29.83 | 3+4=7/10 | No | | No |
|  | 187 | | 65 | | Biopsy | 40.2 | 3+4=7/10 | No | | No |
|  | 189 | | 75 | | Biopsy | 42 | 4+3=7/10 | No | | No |
|  | 31 | | 67 | | Biopsy | 64 | 4+4=8/10 | No | | No |
|  | 73 | | 72 | | Biopsy | 78.5 | 4+3=7/10 | No | | No |
|  | 324 | | 63 | | Biopsy | 4.46 | 3+4=7/10 | No | | No |
|  | 176 | | 90 | | Biopsy | 35.5 | 4+4=8/10 | No | | No |
|  | 151 | | 60 | | Biopsy | 52 | 4+3=7/10 | No | | No |
|  | 24 | | 45 | | Biopsy | 149 | 4+4=8/10 | No | | Yes |
|  | 157 | | 56 | | Biopsy | 28.48 | 3+4=7/10 | No | | No |
| **Validation Cohort (Cohort - 2)** | | | | | | | | | | |
| **BPH***  (N = 16) | 8 | 85 | | Biopsy | | 10 | NA | No | No | |
|  | 15 | 55 | | Biopsy | | 14.29 | NA | No | Yes | |
|  | 20 | 75 | | Biopsy | | 8.03 | NA | No | No | |
|  | 21 | 72 | | Biopsy | | ND | NA | No | No | |
|  | 22 | 72 | | Biopsy | | 27.32 | NA | No | No | |
|  | 59 | 52 | | Biopsy | | 12 | NA | No | Yes | |
|  | 62 | 75 | | Biopsy | | 584 | NA | No | Yes | |
|  | 74 | 56 | | Biopsy | | 12.3 | NA | No | No | |
|  | 77 | 84 | | Biopsy | | 13.6 | NA | No | No | |
|  | 80 | 65 | | Biopsy | | 9.94 | NA | No | Yes | |
|  | 81 | 55 | | Biopsy | | 4.9 | NA | No | Yes | |
|  | 99 | 59 | | Biopsy | | 12.9 | NA | No | Yes | |
|  | 106 | 65 | | Biopsy | | 7.84 | NA | No | Yes | |
|  | 108 | 60 | | Biopsy | | 12.9 | NA | No | Yes | |
|  | 124 | 65 | | Biopsy | | 46.59 | NA | No | No | |
|  | 138 | 75 | | Biopsy | | 10.4 | NA | No | Yes | |
| **PCa#**  (N = 13) | 33 | 70 | | Biopsy | | 145 | 4+3=7/10 | No | Yes | |
|  | 35 | 83 | | Biopsy | | ND | 4+4=8/10 | No | Yes | |
|  | 55 | 65 | | Biopsy | | 36.2 | 4+3=7/10 | No | Yes | |
|  | 319 | 78 | | Biopsy | | 5.1 | 4+4=8/10 | No | - | |
|  | 321 | 61 | | Biopsy | | 44.5 | 3+4=7/10 | No | - | |
|  | 339 | 49 | | Biopsy | | 45.93 | 4+4=8/10 | No | - | |
|  | 283 | 85 | | Biopsy | | 9.069 | 4+3=7/10 | No | - | |
|  | 305 | 64 | | Biopsy | | 36 | 3+3=6/10 | No | - | |
|  | 306 | 54 | | Biopsy | | 563 | 4+3=7/10 | No | - | |
|  | 268 | 67 | | Biopsy | | 56.7 | 4+4=8/10 | No | - | |
|  | 265 | 65 | | Biopsy | | ND | 5+5=10/10 | No | - | |
|  | 263 | 71 | | Biopsy | | ND | ND | No | - | |
|  | 262 | 56 | | Biopsy | | 16.3 | 3+4=8/10 | No | - | |
|  | 257 | 74 | | Biopsy | | 200 | 4+4=8/10 | No | - | |
|  | 207 | 72 | | Biopsy | | 88.7 | 3+3=6/10 | No | - | |

* BPH: benign prostatic hyperplasia; #PCa: prostate cancer; ND: Not determined; NA: Not applicable
