# Supplementary material for: Differential Microbial Signature Associated With Benign Prostatic Hyperplasia and Prostate Cancer
Source: Front Cell Infect Microbiol. 2022 Jul 5;12:894777. doi: 10.3389/fcimb.2022.894777 (PMC9294280; doi:10.3389/fcimb.2022.894777)
Supplement: Supplementary file 7 [file Table_2.docx]

**Table S2: 16S rRNA amplicon sequencing results of discovery cohort.**

| **Sample**  **ID** | **Sample type** | **Total number of reads** | **Number of valid reads** | **Mapped reads in sample** | **Number of reads ignored** | **Unmapped reads in sample** |
| --- | --- | --- | --- | --- | --- | --- |
|  | | | | | | |
| 75 | BPH | 1104261 | 985909 | 777861 | 205075 | 2973 |
| 82 |  | 655220 | 593490 | 522355 | 70949 | 186 |
| 87 |  | 799419 | 711781 | 565010 | 146334 | 437 |
| 89 |  | 927426 | 821217 | 648676 | 172284 | 257 |
| 96 |  | 1008564 | 923559 | 810343 | 112802 | 414 |
| 108 |  | 621837 | 431773 | 304982 | 117306 | 9485 |
| 122 |  | 666469 | 593557 | 502923 | 90387 | 247 |
| 128 |  | 744387 | 667794 | 538580 | 128677 | 537 |
| 117 |  | 967297 | 867868 | 719568 | 147600 | 700 |
| 119 |  | 1066090 | 943040 | 746778 | 195839 | 423 |
| 102 |  | 1101046 | 941933 | 708035 | 233602 | 296 |
| 103 |  | 595204 | 522649 | 411846 | 109921 | 882 |
| 113 |  | 831289 | 719302 | 608339 | 110326 | 637 |
|  | | | | | | |
| 38 | PCa | 750526 | 675616 | 552995 | 121299 | 1322 |
| 183 |  | 552995 | 668015 | 529374 | 138245 | 396 |
| 143 |  | 770199 | 661797 | 520644 | 140498 | 655 |
| 139 |  | 828622 | 715126 | 569660 | 145303 | 163 |
| 58 |  | 819069 | 712565 | 558331 | 152915 | 1319 |
| 154 |  | 580732 | 520573 | 410013 | 110509 | 51 |
| 92 |  | 613668 | 526740 | 402772 | 123491 | 477 |
| 16 |  | 646283 | 567182 | 442556 | 123860 | 766 |
| 135 |  | 832539 | 719065 | 563489 | 155245 | 331 |
| 198 |  | 1041432 | 869728 | 632770 | 236483 | 475 |
| 150 |  | 770008 | 640398 | 501879 | 138519 | 0 |
| 100 |  | 747380 | 641470 | 496633 | 144564 | 273 |
| 180 |  | 955519 | 819680 | 650849 | 168728 | 103 |
| 224 |  | 927275 | 806597 | 627645 | 178409 | 543 |
| 93 |  | 575889 | 494132 | 383517 | 110480 | 135 |
| 333 |  | 658827 | 547308 | 465004 | 80968 | 1336 |
| 206 |  | 572421 | 467036 | 337147 | 129762 | 127 |
| 131 |  | 1008630 | 823885 | 596497 | 225875 | 1513 |
| 202 |  | 982134 | 857194 | 674408 | 182665 | 121 |
| 70 |  | 850251 | 751100 | 578092 | 172855 | 153 |
| 134 |  | 797895 | 648385 | 467132 | 179096 | 2157 |
| 148 |  | 886203 | 767887 | 600892 | 166596 | 399 |
| 327 |  | 807942 | 679251 | 551492 | 126364 | 1395 |
| 141 |  | 864864 | 749455 | 590334 | 158527 | 594 |
| 187 |  | 76752 | 66446 | 52862 | 13584 | 0 |
| 189 |  | 1156959 | 1026356 | 878667 | 147606 | 83 |
| 31 |  | 899393 | 742637 | 592566 | 147169 | 2902 |
| 73 |  | 761567 | 679233 | 549509 | 125687 | 4037 |
| 324 |  | 42875 | 19523 | 11285 | 8238 | 0 |
| 176 |  | 924542 | 748054 | 573167 | 173156 | 1731 |
| 151 |  | 555371 | 474376 | 374691 | 99658 | 27 |
| 24 |  | 81398 | 40254 | 32374 | 7880 | 0 |
| 157 |  | 891663 | 790090 | 663216 | 126620 | 254 |
| **Total** | | 35320332 | 30641026 | 24297758 | 6301956 | 41312 |
