# Supplementary material for: Differential Microbial Signature Associated With Benign Prostatic Hyperplasia and Prostate Cancer
Source: Front Cell Infect Microbiol. 2022 Jul 5;12:894777. doi: 10.3389/fcimb.2022.894777 (PMC9294280; doi:10.3389/fcimb.2022.894777)
Supplement: Supplementary file 8 [file Table_3.docx]

**Table S3. Primers for real time quantitative PCR analyses.**

| **Name of the species** | **Gene**  **Segment** | **Accession ID** | **Primers** |
| --- | --- | --- | --- |
| Human papillomavirus 16 (HPV-16) | E2 | NC_001526.4 | 5’-CAGACGACTATCCAGCGACC-3’  5’-GCAGTGAGGATTGGAGCACT-3’ |
|  | E7 |  | 5’-GACAAGCAGAACCGGACAGA-3’  5’-GAGAACAGATGGGGCACACA-3’ |
| Human papillomavirus 18 (HPV-18) | E6 | NC_001357.1 | 5’-AAGCTCAGCAGACGACCTTC-3’  5’-CCCTCCCCGTCTGTACCTTA-3’ |
|  | E7 |  | 5’-CCGAACCACAACGTCACACA-3’  5’-GGAATGCTCGAAGGTCGTCT-3’ |
| Epstein-Barr virus (EBV) | EBNA3A | NC_007605.1 | 5’-GCCCTGGATGACAACATGGA-3’  5’-CAGGTGGGCATCTTCTGCTT-3’ |
|  | EBER1 |  | 5’-GGAGACGTGTGTGGCTGTAG-3’  5’-TTGACCGAAGACGGCAGAAA-3’ |
| Hepatitis B virus (HBV) | Polymerase | NC_003977.2 | 5’-GATGTGTCTGCGGCGTTTTA-3’  5’-GCAACATACCTTGATAGTCCAGAAGAA-3’ |
|  | X protein |  | 5’-CCCGTCTGTGCCTTCTCATC-3’  5’-GTCGGTCGTTGACATTGCTG-3’ |
| JC Polyomavirus (JCV) | Jvgp4 | [NC_001699.1](https://www.ncbi.nlm.nih.gov/entrez/viewer.fcgi?db=nucleotide&id=9628642) | 5’-GGAGACGTGTGTGGCTGTAG-3’  5’-TGTCATGAGTTGCTTGCCCA-3’ |
| BK Polyomavirus (BKV) | VP1 | [NC_001538.1](https://www.ncbi.nlm.nih.gov/entrez/viewer.fcgi?db=nucleotide&id=9627180) | 5’-CCCTTGGAAATGCAGGGAGT-3’  5’-CATTACCTGGGACTGGGCTG-3’ |
| Human herpesvirus 8  (KSHV) | ORF27 | [NC_009333.1](https://www.ncbi.nlm.nih.gov/entrez/viewer.fcgi?db=nucleotide&id=139472801) | 5’-TGGCTCGAAAGCTTCATCGT-3  5’-GCACAGAAACAGGGCTAGGT-3’ |
| Merkel cell polyomavirus  (MCPyV) | VP1 | [NC_010277.2](https://www.ncbi.nlm.nih.gov/entrez/viewer.fcgi?db=nucleotide&id=733573629) | 5’-GTGCCATCCGTTCTGGAAGA-3’  5’-CGAACACCATGAGGAACCCA-3’ |
| Human T-lymphotropic virus 1 (HTLV-1) | Env | [NC_001436.1](https://www.ncbi.nlm.nih.gov/entrez/viewer.fcgi?db=nucleotide&id=9626453) | 5’-ACTGGACCCACTGCTTTGAC-3’  5’-CTAGCGTGGGAACAGGTGAC-3’ |
| Hepatitis C virus (HCV) | polyprotein;protein F | NC_038882.1 | 5’-GAGCGGTCTACGCCTTCTAC-3’ 5’-ATATGGCGACAGAGTCAGCG-3’ |
| *Cellvibrio mixtus* | endo-beta-1,4-xylanase | [Z48926.1](https://www.ncbi.nlm.nih.gov/entrez/viewer.fcgi?db=nucleotide&id=757808) | 5’-CTCATCAGCCCAAAGCTCCA-3’  5’-CACGGCTTGTTCCGAGGTAT-3’ |
|  | MFS transporter |  | 5’-GTACATCCTGCGGGTCAACA-3’  5’-TGACGCAACTGTCTCTGCAT-3’ |
| *Cupriavidus taiwanensis* | Homospermidine synthase | NC_010528.1 | 5’-CCAGGTGATGCCAACGTCTA-3’  5’-TCGTAAAGTTCGGCCCAGTC-3’ |
|  | FAD-dependent oxidoreductase |  | 5’-CACCTGCTTGTGGTTTTCCG-3’  5’-CCATCTCGACATCACTCCCG-3’ |
| *Prevotella copri* | YgiQ family SAM protein | NZ_GG703852.1 | 5’-CTCCAGCGCCAAGACTCAGA-3’  5’-ACCCTTGAAACTGCCACCCA-3’ |
|  | Pyruvate:ferredoxin  oxidoreductase |  | 5’-AGAAGGGTGAGAAGGCGTTG-3’  5’-CCAGAGCAAGCACCAGAGAA-3’ |
| *Propionibacterium acnes* | RuvX | NC_006085.1 | 5’-GGAAAAGCACGGATTGGCGT-3’  5’-ATAGCCTCGGTCAACTGGCG-3’ |
|  | TIGR03767 family metallophosphoesterase |  | 5’-CAGCAGTCCCCAGTCAGAAG-3’  5’-GGTTTTCCCCAGGTAGACGG-3’ |
| *Cupriavidus campinensis* | Acetyl-CoA C-acyltransferase | FGG12_RS01630 | 5’- CGAGATGTTGTAGCGCTTGG -3’  5’- GGAGATGAACCGCCACATGA -3’ |
|  | Isochorismatase family protein |  | 5’- GGCAAAGCCGTTCACGAAAT -3’  5’- TGCATGAGGAAGTCCAGACC -3’ |
| *Kocuria palustris* | ABC-F family ATP-binding protein | NZ_CP012507.1 | 5’-GGCCTGAAGCTCGGCTACTT-3’  5’-GGCCAGTCGGGTCTTCTCAC-3’ |
|  | Glutamate synthase large subunit |  | 5’-GTCATGCCGTACTTCGTCCA-3’  5’-AACGACGAGCAGTAGATGCC-3’ |
| *Methylobacterium organophilum* | Methanol dehydrogenase alpha subunit-like gene | EF562471.1 | 5’-TTTGCGGCACCCAACTG-3’  5’-CCGAAGATCGTCATCGTCCA-3’ |
|  | Formyltransferase/hydrolase complex subunit D |  | 5’-CCTCGACTAACGACGCCTAC-3’  5’-GTAGTTGCCGGCCGTGAC-3’ |
| Human | Glyceraldehyde 3-phosphate dehydrogenase (GAPDH) | NG_007073.2 | 5’-AGCTGGCCCGATTTCTCCTC-3’  5’-ATGACTCAGCTTCTCCCGGC-3’ |
